# Reliability of the Mid Upper Arm Circumference for the Assessment of Wasting among Children Aged 12-59 Months in Urban Ibadan, Nigeria

**Published:** 2012-06

**Authors:** M. D. Dairo, Modupeoluwa E. Fatokun, Modupeoluwa Kuti

**Affiliations:** 1 *Department of Epidemiology and Medical Statistics University of Ibadan, Nigeria;*; 2 *Department of Chemical Pathology, University College Hospital, Ibadan, Nigeria*

**Keywords:** malnutrition, wasting, sensitivity, specificity, reliability, mid upper arm circumference

## Abstract

Although the mid upper arm circumference (MUAC) is used as a proxy to assess wasting in children, its validity abounds in controversies. This study therefore assessed the validity of MUAC as a measure of nutritional status among children aged 12-59 months in urban setting in western Nigeria. A cross sectional study of children aged 12-59 months in selected nursery schools in Moniya, Akinyele local government area, Oyo state was carried out between February to April 2010. The age, weight, length and MUAC, were obtained from the school children who were selected through total sampling. The sensitivity, specificity, positive predictive value, and negative predictive values of the MUAC were determined using weight for age as gold standard (underweight). Z-Scores for anthropometric indicators were calculated using EPI-NUT programme. Receivers operating curve was prepared to determine the optimal cut off of MUAC for the sensitivity and specificity. The overall prevalence of under nutrition among the respondents was 5.6%. The mean mid upper arm circumference was 15.47 ± 1.4 cm and appears to increase with age from 11.49 ± 3.0 at 12-23 months and peaks at age five with a value of 18.05 ± 3.5. At 13.5 cm cut off, the sensitivity of MUAC is 20%, and the specificity is 95.3%, with a Kappa of 16.7%. The receivers operating curve reveals an optimum cut off of 15.5 cm with optimal but improved MUAC sensitivity of 80% and specificity of 53.5%. Thus MUAC was a poorly sensitive indicator of under nutrition at a cut-off below 13.5 cm but highly sensitive at 15.5 cm. A higher cut off value is therefore for recommended for screening for acute malnutrition among under five children.

## INTRODUCTION

The prevalence of under-nutrition in developing countries is very high. In Latin America, the prevalence of wasting ranges between 19-21% while stunting was about 20-25%. West Africa has prevalence rates of 17.4% while in Nigeria, some authors have documented a rate as high as 27.5% (1). The measurement of MUAC has become widely used in the assessment of the nutritional status of children, due mainly to its relative independence of age in one to five year-old children; however, the age-independence of MUAC has been disputed (2). Although it is reported that MUAC can be used as a proxy of wasting-weight for height, the two indices disagree over which children are at risk. The agreement between MUAC and weight for age appears to vary within different groups of children (3), and it is unclear which may prove to be the most adequate predictor of clinically diagnosed malnutrition. Though MUAC of <12.5 and <13.5 cm has been used to detect severe and moderate malnutrition respectively, the standard against which nutritional status of a sampled population should be determined has been controversial (4). The MUAC is relatively easy to perform and requires a minimal amount of time and measurement. The MUAC has been used as a measure of malnutrition, its validity in tropical regions such as Nigeria has however not been reported in available literature. This study intends to determine and document the validity of MUAC, and its usefulness in detecting the extent of malnutrition among under- five children in Nigeria.

## MATERIALS AND METHODS

### Study area

The study was conducted at Moniya, Akinyele local government, Oyo State. Ibadan is made-up of five (5) urban Local Governments of Ibadan North, Ibadan North East, Ibadan North-West, Ibadan South-East and Ibadan South-West and six (6) Sub-urban Local Governments of Akinyele, Egbeda, Ido, Lagelu, Oluyole and Ona-Ara. Akinyele local government is divided into eight major zones .i.e. Moniya, idi-ose, Orogun, Ojoo, Akinyele, Ijaiye, Sasa, and Akingbile. Moniya is the seat of Akinyele local government and the people are predominantly butchers, traders in cocoa, kolanut and cattle. The target population are children aged 12-59 months. Those children who are ill at the time of the study or chronically ill were excluded from the study. Five nursery schools were randomly selected from the list of registered nursery schools in Moniya, Akinyele local government. A total sampling of under-five children, present at the time of the study in all the selected nursery schools was carried out.

### Study design

The study design was a descriptive cross sectional survey.

**Data collection.** An interviewer administered data collection form was designed into which data such as age, weight, length, arm circumference, medical history and immunization history of each child was recorded. Measurements of MUAC (to the nearest 1.0 cm) were made using a non-stretch tape measure, nude weight of the child (to the nearest 0.1 kg) by a Salter bathroom scale and Supine length (to the nearest 0.1 m) was measured with a Holtain infantometer. Age was determined (calculated in the nearest months) by asking of both the child’s age and date of birth. Data was collected by the principal investigator and two trained research assistants.

**Data management.** Data cleaning and editing of data collection form was done on daily basis. Data was analyzed using EPI-INFO computer package and statistical package for social sciences. The Z-scores for different nutritional indices, weight-for-age, height-for-age and weight-for-height were calculated in reference to WHO standards by using EPI-INFO programme. The sensitivity, specificity, positive predictive value, and negative predictive values of the MUAC was determined using weight for age, and weight for height indicators as gold standard respectively. The following anthropometric indicators were determined: weight for height (wasting); weight for age (underweight) and height for age (stunting). Descriptive analysis was used in summarizing demographic data. The chi-square test was used to evaluate association between categorical variables. The analysis was considered to show significant associations when the p value was less than 0.05. A receiver’s operating curve of MUAC and weight for age was plotted and hence, a more sensitive cut- off for MUAC was determined.

**Ethical issues.** Ethical approval was sought and obtained from the UCH/UI ethics review committee. Informed consent was sought from the proprietors of the five nursery schools used and also the parents of each participant in the language they understood. It was made known to the participants’ parents and the school authority that they reserved the right to withdraw the respondent’s participation at any time they wish. Data was treated with absolute confidentiality.

## RESULTS

A total of 319 children were studied out of this, 49.8% were male and 50.2% of them were female with a mean age of 39.63 ± 11.98 months. Table 1 shows the age distribution and gender of the respondents respectively. The mean mid upper arm circumference in the Nigerian under five year old was 15.5 ± 1.4 cm and ranges between 9 cm to 20 cm (Table 2). The mean MUAC progressively increases with age and this difference is statistically significant. The overall prevalence of under-nutrition by MUAC is 5.6%. Of the under-nourished respondents, 66.7% were females and 33.3% were males (Table 3).

**Table 1:** Age and gender distribution of participants

AGE GROUPS(MONTHS)	FREQUENCY	PERCENTAGE

12-23	38	11.9
24-35	79	24.8
36-47	99	31
48-59	103	32.3
Total	319	100.0
GENDER		
Male	159	49.8
Female	160	50.2
Total	319	100

**Table 2:** Summary statistics of respondents’ anthropometric variables

Anthropometric variables	Mean ± SD	Minimum	Maximum

Weight of respondents	15.53 ± 3.9 kg	7.0 kg	26.0 kg
12-23 months	11.49 ± 3.0 kg	7.0 kg	22 kg
24-35 months	14.33 ± 2.8 kg	8.5 kg	22 kg
36-47 months	15.39 ± 3.4 kg	10.0 kg	26.0 kg
48-59 months	18.05 ± 3.5 kg	10.0 kg	26.0 kg
MUAC	15.47 ± 1.4 cm	9.0 cm	20.0 cm
12-23 months	13.86 ± 1.8 cm	9.0 cm	17.0 cm
24-35 months	15.42 ± 1.4 cm	10.0 cm	18.0 cm
36-47 months	15.74 ± 1.2 cm	12.5 cm	18.5 cm
48-59 months	15.84 ± 1.1 cm	14.0 cm	20.0 cm

**Table 3:** Sex-specific prevalence of under-nutrition by mid upper arm circumference

Gender of respondents	Undernourished (<13.5 cm)	Normal (≥13.5 cm)	Total

Male	6 (3.8%)	153 (91.2%)	159 (100%)
Female	12 (7.5%)	148 (92.5%)	160 (100%)
Total	18 (5.6%)	301 (94.4%)	319 (100%)

The sensitivity of MUAC using weight for age as gold standard is 27.5%, and the specificity is 96.8%. The extent to which the agreement between MUAC and weight for age improves on chance agreement is 28.6% (Kappa agreement) Table 4. The sensitivity of MUAC using weight for height as gold standard is 20%, and the specificity is 95.3%. The extent to which the agreement between MUAC and weight for height improves on chance agreement is 16.7% (Kappa agreement) Table 5. The sensitivity of MUAC using height for age as gold standard is 30.4%, and the specificity is 96.6%. The extent to which the agreement between MUAC and Height for age improves on chance agreement is 32.3% (Kappa agreement) Table 6.

**Table 4:** Validity of MUAC using weight for age as gold standard

MUAC	Underweight		Total
	Positive	Negative	

Positive	9	9	18
Negative	26	275	301
Total	35	284	319

Sensitivity=25.7% and specificity=96.8%, Kappa=28.6% (negligible agreement).

**Table 5:** Validity of MUAC using weight for height as gold standard

MUAC	Wasting		Total
	Positive	Negative	

Positive	4	14	18
Negative	16	285	301
Total	20	299	319

Sensitivity=20%, Specificity=95.3%, Kappa=16.7% (negligible agreement).

**Table 6:** Validity of MUAC using height for age as gold standard

MUAC	Stunting		Total
	Positive	Negative	

Positive	7	11	18
Negative	15	286	301
Total	23	296	319

Sensitivity=30.4%, Specificity=96.6%, Kappa=32.3% (negligible agreement).

**Figure 1:**
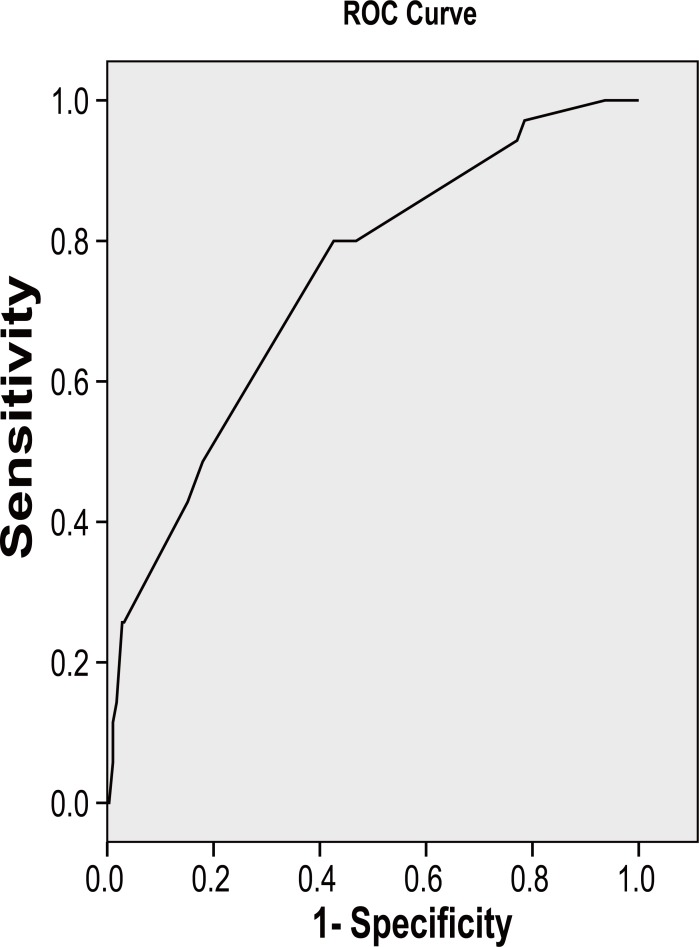
Receiver’s operating curve of MUAC against weight for age. The ROC shows an optimal cut off of 15.5cm with specificity of 53% and sensitivity of 80%.

A receiver operating curve was plotted to determine the optimal cut off and sensitivity and specificity for the MUAC, and was found to be a sensitivity of 80% and specificity of 53.5%. At these optimal reliability values, the cut off for MUAC is 15.5 cm (Table 7) (Figure 1).

**Table 7:** Table showing the validity of MUAC using weight for age at different cut-offs

Cut-offs	Sensitivity (%)	1-Specificity (%)

11.0	5.7	1.1
12.25	11.4	1.1
12.75	14.3	1.8
13.25	25.7	2.8
13.75	25.7	3.2
14.25	42.9	15.1
14.75	48.6	18.0
15.25	80.0	42.6
15.75	80.0	46.8
16.25	94.3	77.1
16.75	97.1	78.5

## DISCUSSION

Mid-upper arm-circumference has been considered a valid and simple screening measure for protein-energy malnutrition in children between 1 to 5 years of age. In this study, the sensitivity and specificity of MUAC in diagnosing under-nutrition at cut off level of <13.5 cm were 20% and 95.3% for wasting; 25.7% and 96.8% for underweight; and 30.4% and 96.6% for stunting. This result could be explained in the light of the fact that the prevalence of under nutrition in the study area was very low, probably because the study was school- based.

As shown in the results above, the agreement between the MUAC as an assessment tool for different spectrum of malnutrition is very poor. The agreement of 16.7% between the weight for height and MUAC at the 13.5 cm cut off in particular suggests the need to determine a different MUAC cut off criteria for determining acute malnutrition (wasting). As shown in the study, an optimal cut-off of 15.5 cm yielded a sensitivity of 80% and specificity of 53%. This might be a better cut-off as it is has a higher sensitivity and will therefore detect majority of those with malnutrition and yield better results from field studies. The 15.5 cm cut off is therefore recommended for adoption in Nigeria and further studies on the reliability and desirability of this new cut off is suggested.

In conclusion, a cut-off of 15.5 cm for MUAC and is recommended for field screening for under nutrition in children in south-western Nigeria and similar areas. Further studies with larger sample size is recommended to confirm this finding.
